# Investigating long‐term, progressive cerebral microvascular alterations in late‐onset Alzheimer's disease using APOE4 transgenic mice

**DOI:** 10.1002/alz.090063

**Published:** 2025-01-09

**Authors:** Jang‐Hoon Lee

**Affiliations:** ^1^ Brown University, Providence, RI USA

## Abstract

**Background:**

Recent studies show apolipoprotein E4 (APOE4), the strongest known genetic risk factor for late‐onset Alzheimer’s disease, is associated with vascular dysfunctions such as blood‐brain barrier breakdown, in both animal models and humans. However, there is a lack of understanding on how vascular alterations progress with age in APOE4.

**Method:**

Human APOE4 (m/f, n=10 per group) and APOE3 targeted replacement mice (m, n=10) were used to investigate temporal dynamics of long‐term, progressive cerebral microvascular alterations in APOE4. we acquired a set of imaging data including optical coherence tomography (OCT) angiogram and doppler OCT to quantify relative changes in pial and penetrating vessels with age (starting at 13 weeks of age (WOA) and repeated every 4 weeks until 57 WOA). For assessing cognitive function, novel object location test (NOL) was used and performed every 4 weeks starting from 17 WOA.

**Result:**

We compared the rate of change with age (RCA) values between the groups to find the effects of APOE4 and female. In pial vessel diameter, both APOE4‐m and APOE4‐f groups showed vessel diameter decreasing with age at a rate of 2.7 and 3.1% per month, respectively. In contrast, the control group (APOE3‐m) exhibited a slight increase in vessel diameter at a rate of 1.0% per month. When compared between groups based on RCA values, the age of significance (AOS) for the effect of APOE4 was 28 WOA. Adding the effect of female with APOE4, the result showed an earlier AOS of 26 WOA. In penetrating vessels (diameter/flow change in arterioles/venules), a similar trend was observed with both APOE4 groups exhibits more rapid decreasing RCAs and earlier AOS (27 WOA as earliest) than APOE3. More importantly, these AOS values were much faster than cognitive decline (51 WOA).

**Conclusion:**

We found vascular alterations in both pial and penetrating vessels occurred earlier than cognitive decline, being generally greater and earlier in female APOE4 than male APOE4 mice. This female effect in APOE4, although the underlying mechanisms behind such finding is beyond the scope of this study, could still provide valuable insights on understanding vascular dysfunction in sex‐dependent manner.

